# Orchestrating neuroimaging data processing using the ‘Snakemake’ workflow manager

**DOI:** 10.1007/s00429-026-03189-3

**Published:** 2026-09-04

**Authors:** Damien J. Mannion, Maria del Mar Quiroga, Jacob M. Paul, Marta I. Garrido

**Affiliations:** 1 Melbourne Data Analytics Platform (MDAP), The University of Melbourne, Parkville, Australia; 2 Melbourne School of Psychological Sciences, The University of Melbourne, Parkville, Australia; 3 Graeme Clark Institute for Biomedical Engineering, The University of Melbourne, Parkville, Australia

**Keywords:** Workflow, Data processing, Data analysis, Infrastructure, Computing

## Abstract

The processing of neuroimaging data typically involves a complicated set of operations, which often require different software packages and have intensive computational and storage demands. Although there are many options available for the neuroimaging researcher to establish their preferred set of processing operations, there is relatively little guidance on how to orchestrate such operations within a coherent data processing workflow. Here, by describing the construction of a simplified neuroimaging processing workflow, we demonstrate how the established general-purpose workflow manager ‘Snakemake’ can be used to facilitate neuroimaging data processing. Snakemake allows researchers to use a Python-based markup language to describe how input files are transformed into output files. Snakemake then uses these rules to schedule and execute the computational jobs that are required to generate the output files, without unnecessary recreation of existing output files. This allows the complete processing workflow to be executed with a single command and creates workflows that are interpretable, efficient, and reproducible.

A neuroimaging researcher typically needs to run a complicated chain of operations to go from the raw data to the final outcomes of their analysis. These operations often encompass multiple software packages, which often operate in different programming languages, and require intensive processing on large quantities of data. Fortunately, there is a rich and comprehensive ecosystem of software and educational material for researchers to implement such operations and to develop the ingredients of their own processing workflows. However, there has been comparatively little attention given to the oversight of how and when such operations are executed: the implementation and management of an overall workflow.

In a typical approach, the workflow consists of a series of sequential executions in which an ordered set of commands or operations are launched manually by the researcher. Researchers often increase the automation of such a workflow by writing custom scripts to iterate over repetitive components, such as participants and runs, in stages like pre-processing. Some researchers also increase the sophistication of the workflow by adding functionality like parallelisation to speed up computation and by expanding the scope to incorporate custom analyses and visualisation to form a complete end-to-end workflow for a particular project. They may then implement abstraction over the specific project, providing capacity for the workflow management to be applied to new analysis requirements—and, with public release, potentially by others outside of their research group.

The primary downsides of these custom approaches stem from either their dependence on manual actions (and the associated high labour and time demands) or the need for implementation of complex workflow management tasks. These factors can increase the vulnerability of the workflow’s integrity due to the chances of operational inconsistencies (e.g., forgetting to change all instances of a participant identifier when running a command) and workflow inconsistencies (e.g., processing a subset of participants with unintentionally different settings to the others). Outcomes from such approaches can be also difficult to reproduce due to the workflow’s dependence on custom procedures and an absence of mechanisms to support functionality beyond the user’s current machine and environment; this is particularly relevant given that journals are beginning to require their published articles to be demonstrably reproducible by a third party for acceptance (e.g., Hardwicke and Vazire, 2024). Finally, custom workflow managers can also be difficult to maintain and accrue features due to their typical dependence on a small set of developers and the priorities and approaches of individual research groups.

An alternative is to instead use established general-purpose workflow management software to implement neuroimaging processing workflows. For example, ‘Snakemake’ (Köster and Rahmann 2012; Mölder et al. 2021) is an open-source workflow manager that has its origins in scientific data analysis and has particularly well-established usage in the biosciences (Mölder et al. 2021). Using Snakemake to manage neuroimaging processing workflows has three key advantages. First, it provides ease of reproducibility by allowing the entire workflow to be executed using a single command, with adaptability to different computational environments (e.g., different amounts of available RAM or running on a high-performance computing platform) and storage providers (e.g., local or remote storage). Second, it is efficient due to its automatic parallelisation (e.g., processing steps can often run in parallel over participants and runs) and its ability to limit operations to those that are necessary to maintain consistency between the workflow description and its output (e.g., a change to a parameter affecting late stages of the workflow does not require re-running earlier stages of the workflow). Third, it promotes understandability by expressing workflows in a standard language that provides both the constraints that enhance readability and comprehension (e.g., by providing a common framework) as well as the flexibility to accommodate the diversity of operations found within neuroimaging workflows (e.g., permitting the use of different software packages and programming languages).

There are many general-purpose workflow managers that provide the benefits of workflow management in science (Perkel 2019). However, there are key features and attributes that make Snakemake a particularly appealing option for the neuroimaging researcher. Because the Python programming language is the foundation of Snakemake, it is likely to be more approachable than other options that use languages that are less common in neuroimaging. Additionally, because Snakemake incorporates Python within a domain-specific language, it requires less advanced Python knowledge than workflow managers that involve specifying a workflow completely in Python. Furthermore, its origins in scientific workflow management provide Snakemake with a feature set and approach that is more likely to match the requirements of a neuroimaging researcher than an option with a more generic focus. Finally, Snakemake has a large user and developer base, a long development history, excellent documentation and learning resources that are constructed for a scientific audience, and widely-available examples of workflows across many disciplines. For a comparison with alternative general-purpose workflow managers, we recommend the Snakemake authors’ perspective on the positioning of Snakemake (Mölder et al. 2021), a detailed evaluation of four general-purpose workflow managers in the implementation of a scientific workflow (Bountris et al. 2026), and a tutorial on the use of the Nextflow workflow manager (Di Tommaso et al. 2017) in a neuroimaging context (Bollmann 2026).

There are also workflow managers that are specifically dedicated to neuroimaging processing, such as fMRIPrep (Esteban et al. 2019), afni_proc.py (Reynolds et al. 2024), and Nipype (Gorgolewski et al. 2011). Such software is typically targeted at a specific subset of a complete processing workflow and is hence well-suited to being integrated within an overarching Snakemake workflow, rather than being a competing alternative. For example, functional image pre-processing could be delegated to fMRIPrep in a single step in a Snakemake workflow. Other Snakemake steps can then implement additional aspects of a neuroimaging processing workflow such as group-level analysis, calculation and reporting of descriptive and inferential statistics, generation of visualisations and figures (our accompanying website provides an example of a Snakemake workflow that incorporates a step that creates a custom figure from pre-processed data^1^), analysis of data from other imaging modalities or from behavioural tasks, and the compilation of research outputs such as reports and manuscripts. Hence, by incorporating such neuroimaging-focused functionality within a general-purpose workflow manager, the benefits of the neuroimaging-focused workflow implementation are preserved while the scope of the research project that is under workflow management is considerably expanded—allowing the identified benefits of Snakemake, such as reproducibility, to encompass a broader range of research components.

Here, our goal is to describe the operation of Snakemake and outline how it can assist with the challenges of neuroimaging processing.^2^ We consider the foremost barrier to adopting Snakemake within neuroimaging is a lack of awareness of its potential benefits. Additionally, the primarily declarative nature of Snakemake, in which a workflow is described as a set of ‘rules’ which are then converted into jobs that are scheduled and executed by Snakemake (consistent with its inspiration from the Make software; Feldman, 1979), can be conceptually challenging to new users who are more familiar with the more imperative approach of a manual or scripted command runner.

We aim to reduce these barriers by conveying the conceptual foundations of Snakemake operation in a neuroimaging context. To do so, we will reference an implementation of a basic workflow (available on Github^3^) that processes data from two runs of functional imaging and an anatomical acquisition from each of three participants in the study by Poldrack et al. (2016). We chose this study because its raw data are readily available online (Bilder et al. 2020) and it used relatively straightforward structural and functional MRI acquisitions from a frequently-used magnet strength (3T). The goal of this example workflow is to produce motion-corrected BOLD images that are coregistered with the anatomical image for each participant, using AFNI software (Cox 1996; Cox and Hyde 1997; Saad et al. 2009). This workflow is relatively simple and is not intended to promote a particular approach or best-practice for fMRI data processing itself, or to describe and explain the AFNI commands that comprise the workflow, but instead to position the operation of Snakemake and its key features within a domain-relevant context without being burdened by domain-specific details.

The conceptual focus of this paper is supplemented by an accompanying website^4^ that provides a detailed step-by-step workthrough of the construction of a Snakemake workflow. This workthrough elaborates the example described here with additional stages, documents a strategy for converting a processing step into a workflow stage, and demonstrates the process of incremental workflow construction.

## Conceptual overview

The foundation of our example project is an existing set of raw data that consists of two functional MRI images (corresponding to two different tasks) and an anatomical MRI image, for each of three different participants. Our knowledge of neuroimaging data processing tells us that we can obtain the desired workflow outcomes by beginning with such files and executing three computational steps: motion correction, temporal averaging, and coregistration.

**Fig. 1:**
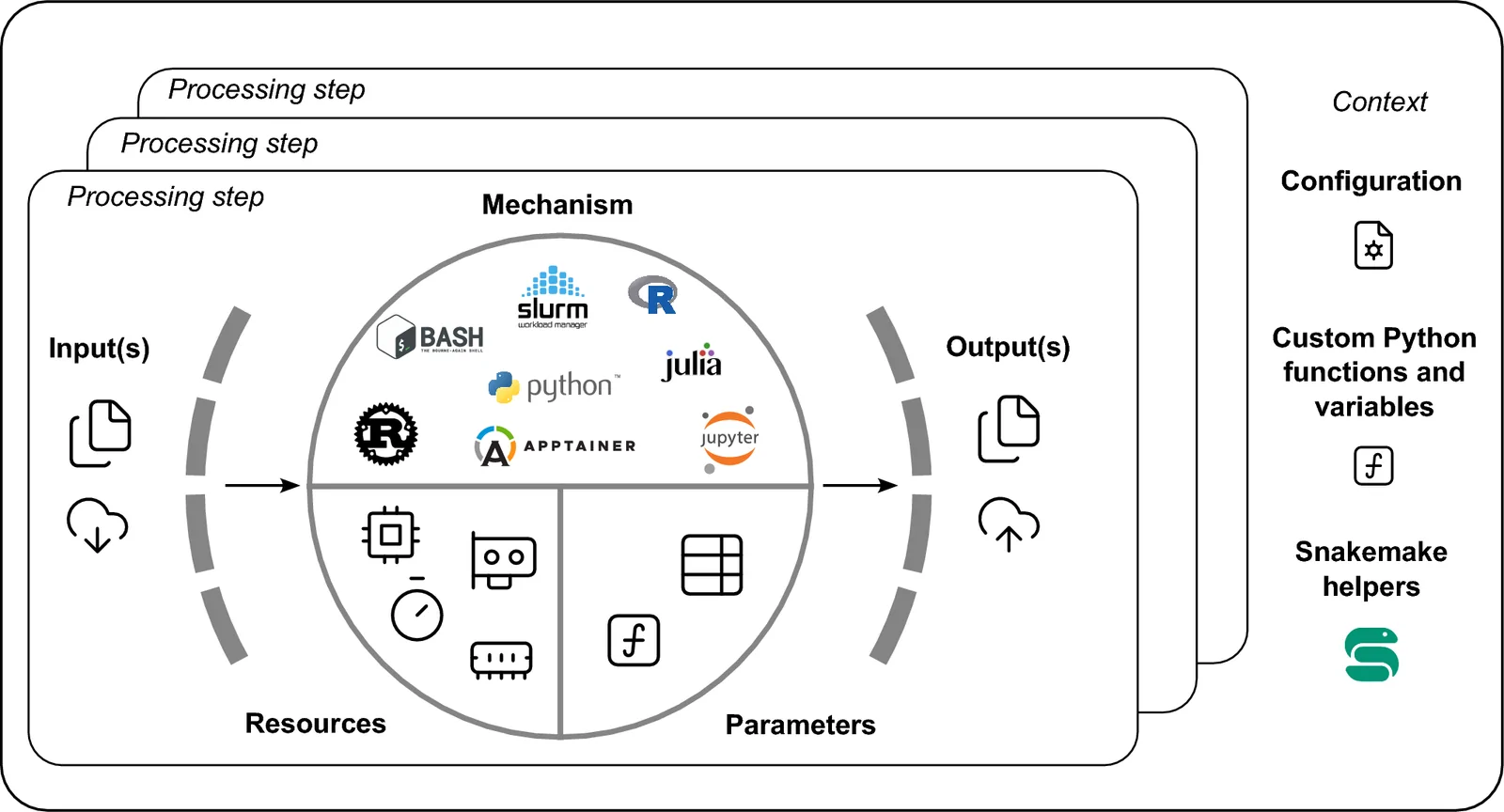
Diagram of the conceptual organisation of key components in a Snakemake workflow

The conceptual structure of a processing step in the Snakemake workflow manager is depicted in Fig. 1. For a given step, we can first think about the changes to a filesystem that are produced from the execution of the step (the *outputs*). We can then think about the required information, obtainable from files, for producing the outputs (the *inputs*). The process that transforms between input and output is the *mechanism*, which consists of shell commands or code (in languages such as Python and R) and the execution environment (such as containers and high-performance computing clusters). Such transformation requires *resources*, such as quantities of RAM and availability of hardware such as a GPU, and can have *parameters* that modify the operation of the transformation. Finally, the step is executed in a context where it can access global configuration values, user-defined functions and variables (in Python), helper functionality that is provided by Snakemake, and details of other steps in the workflow.

Our goal is now to translate the conceptual knowledge of the processing steps into the domain-specific language and conventions of Snakemake. To do so, we will first set up the Snakemake project and then work through the implementation of the steps (each called a ‘rule’) using the conceptual framework described above. To aid in the translation into Snakemake, Table 1 provides a glossary of key Snakemake terms. We will then consider the execution of the workflow with respect to the goals of the current analysis.

**Table 1:** Glossary of key Snakemake terms and their definitions

Term	Definition
collect	An alias of expand; useful as a semantic indication when gathering paths from other rules
configfile	A directive that specifies the location of a configuration file (e.g., "config/config.yaml"); its contents are then available as a Python dictionary within the Snakefile and the rules
container	A rule directive for specifying the path of an Apptainer container image that can be used when executing the rule. The value is any URL that is supported by Apptainer, such as filesystem or Docker Hub locations
Directive	A keyword that contains values relating to the keyword (e.g., output)
envvars	A directive containing values that describe the name(s) of environment variables that are required to be specified before workflow execution (e.g., MLM_LICENSE_FILE)
expand	A Snakemake helper function that resolves brace-contained identifiers (e.g., wildcards) with user-provided values
input	A rule directive for specifying the files that are required for the execution of the rule. Typically specified as a set of key=value pairs, where values can be strings (optionally containing wildcards) or input functions
Input function	A Python function that accepts a wildcards argument (containing the current value of each wildcard in a rule) and returns paths to use as input in a rule
log	A rule directive for specifying one or more paths to be used as log files during rule execution; must contain any wildcards that are present in the output directive
output	A rule directive for specifying the changes to the filesystem that are caused by the execution of the rule. Typically specified as a set of key=value pairs, where values are strings (optionally containing wildcards)
Parameter function	A Python function that accepts a wildcards argument (containing the current value of each wildcard in a rule) and returns a value to use as a param value in a rule. Can optionally take additional arguments (input, output, threads, resources), which contain the state of directives in the rule at the time of the rule execution
params	A rule directive for specifying parameter values for use in the rule. Specified as a set of key=value pairs, where values are Python variables or parameter functions
resources	A rule directive for specifying the resources that are used by a given execution of the rule. Specified as a set of key=value pairs, where keys include mem (RAM usage), runtime (execution time), disk (the required amount of disk space), gpu (the number of GPUs required), and tmpdir (the location for temporary storage)
rule	A directive for the specification of a step in the workflow. Has a user-defined name and contains a series of directives. Typically contained in a single file per rule, with a .smk extension
rules	A Python variable that contains information about the rules in a workflow; typically used to reference the output of other rules
script	A rule directive for specifying the location of a file, relative to the location of the rule definition file (typically stored in ../scripts/). Can be written in languages such as Python and R, which have access to an injected variable that exposes the information contained within the calling rule (e.g., the values of input and output directives) to the script
shadow	A rule directive for specifying that the rule be executed in an isolated temporary location, with potential values indicating the method for file availability in the temporary location (e.g., "shallow")
shell	A rule directive for specifying a shell command to run during the rule execution. Can contain references to information contained within the calling rule (e.g., the values of input and output directives)
Snakefile	The entry point for Snakemake workflow execution; typically located at workflow/Snakefile and containing include directives to import rule definitions and a target rule
subpath	A Snakemake helper function for extracting elements of path strings (e.g., a parent directory)
threads	A rule directive for specifying the number of cores that are allocated to a job that is executed from the rule
Wildcard	A braces-contained identifier within a path string that acts as a placeholder for variable content (e.g., "{sub_num}"). The wildcard is replaced with a specific value as required during workflow execution

## Setup

We begin by installing Snakemake into our computing environment. Because Snakemake is a Python package, this can be done via a Python project manager (recommended) such as uv, via pip in a Python virtual environment, or via a system package manager. We recommend that the version of Snakemake that is installed be documented (we are using version 9.9.0 in this article). We also recommend that the ‘Apptainer’ software (formerly known as ‘Singularity’; Kurtzer et al., 2017; Singularity Developers, 2021) be installed; this provides the capacity for executing commands within containers (to be described further below), and is typically installed using the system package manager. The operating system provided by the Neurodesk project^5^ (Renton et al. 2024) is a convenient option for accessing both Snakemake and Apptainer with minimal setup requirements (see Doerig, 2026, for a tutorial notebook on using Snakemake with Neurodesk).

**Fig. 2:**
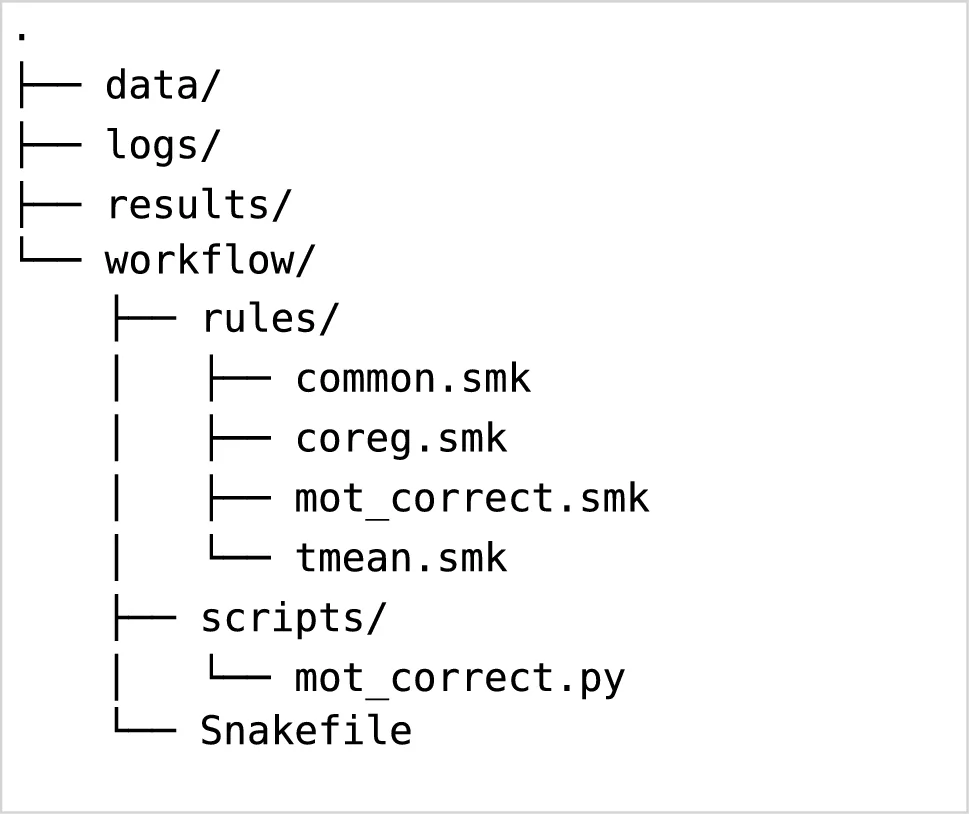
Key components of the workflow directory structure for the example workflow. Note that this shows the contents prior to the data directory being populated with the raw data, and omits additional supportive structure that would typically be present within the workflow directory (e.g., README.md, pyproject.toml, .git, LICENSE, etc.)

We then prepare the filesystem for the Snakemake project, which typically follows a standard structure. We first designate a directory to contain the project-relevant files; we recommend that this directory be the root of the repository of a version-control system (e.g., git). Within this root directory, we then create two sub-directories: data (to contain the raw data)^6^ and results (to contain the outputs from the workflow). Note that these two sub-directories should be excluded from version control. We then create an additional sub-directory called workflow. Within this sub-directory, we create additional sub-directories named rules and scripts and a plain-text file called Snakefile; the purpose of these components will be elaborated below. A diagram of the structure of the key components of the workflow directory is shown in Fig. 2.

## ‘Rules’

The ‘rule’ is the key foundational concept in Snakemake, and the bulk of implementing a workflow in Snakemake involves specifying a set of rules. Each rule describes the production of a set of *outputs*, where an output is defined as a change to a filesystem (typically the creation of a new file). The rule forms a recipe that specifies the *inputs* (the files on which the production of the output depends), the *mechanism* (the software that executes to produce the output given the information contained in the rule), the *parameters* (the configuration settings that affect the operation of the mechanism), and the *resources* (the computational requirements for executing the rule). Each rule is given a user-defined identifier and is specified using a domain-specific language that resembles YAML and incorporates Python.

**Fig. 3:**
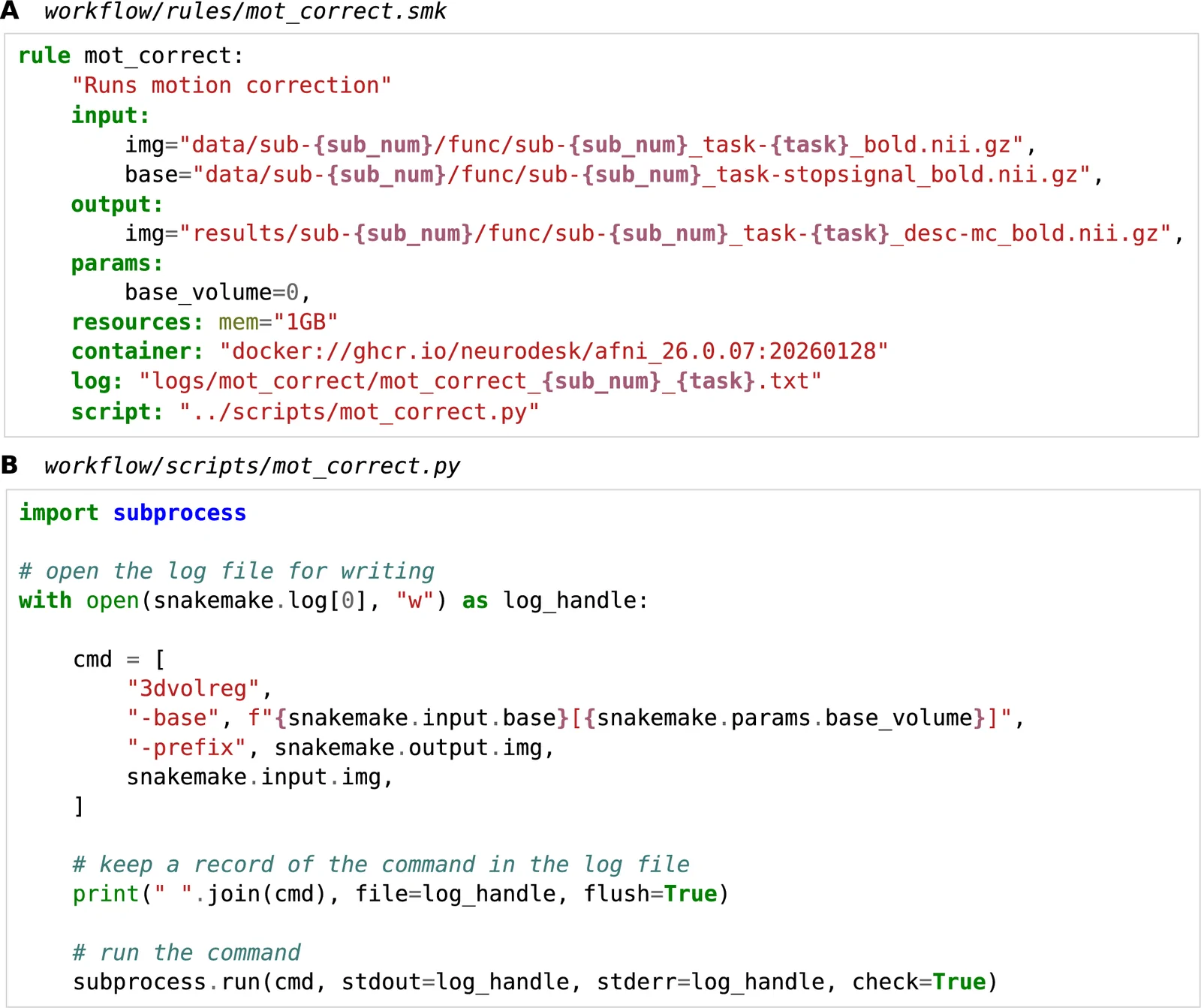
The rule (**A**) and script (**B**) for the motion-correction step in the example workflow

**Fig. 4:**
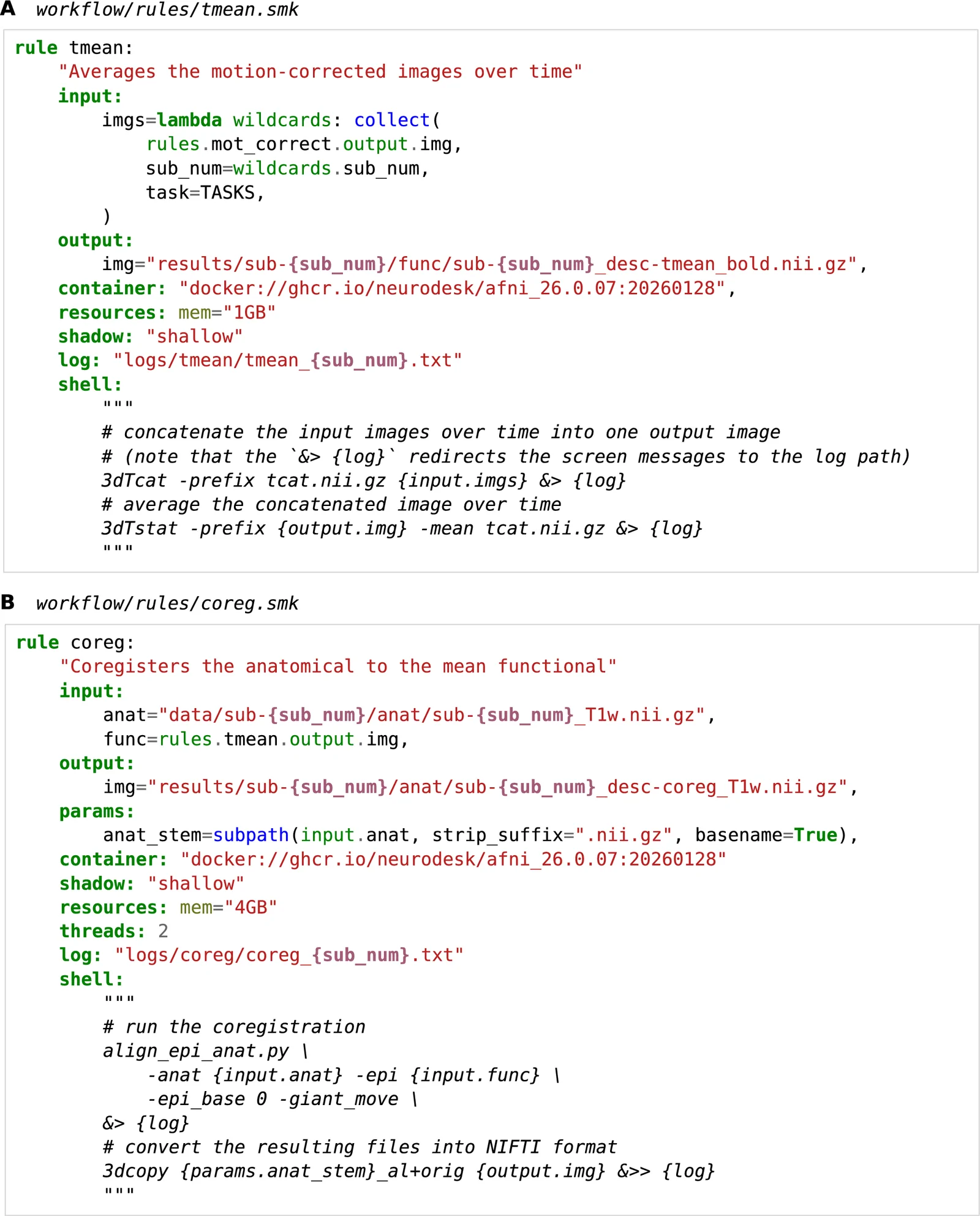
The rules for the temporal averaging (**A**) and coregistration (**B**) steps in the example workflow

For our example project, we will need to write a rule for each of the three processing steps (motion correction, temporal averaging, and coregistration). The rules are contained within separate files, each with a .smk extension, within the rules directory described previously. The contents of these rules are shown in Figs. 3 and 4; below, we will work through the key aspects of the construction of these rules.

### Outputs

The outputs of a rule are listed as items within the output directive. Each output is specified as a string describing a path on a filesystem that is affected by the execution of the rule, and can be given a custom name that can be used to reference the path. The paths are typically specified as relative locations beginning with results/, which are then converted to absolute paths by Snakemake (using the current working directory as the base for the absolute paths, by default) when executed. These strings can either be specified literally or via the return value of a Python function.

The dependence of paths on specific details (e.g., the inclusion of a specific subject number in the name of a directory or file) is alleviated in Snakemake by the use of *wildcards*. A wildcard is an identifier within braces that is contained within the path string (e.g., "{sub_num}"), which acts as a placeholder in the path specification and allows rules to generalise across different values of the wildcard. Conceptually, this can be thought of as the rule indicating that its execution produces output paths that follow a particular *pattern*; alternatively, and more in the spirit of Snakemake, it can be thought of as a given path being able to be produced by the rule if the wildcards are set to particular values.

For our example workflow, the output paths for each of the rules contain wildcards for the subject identifier (sub_num). Note that the rule for motion correction (Fig. 3A) has an additional wildcard for the functional imaging task type (task). The inclusion of this additional wildcard is because the motion correction operates independently across the two functional imaging runs (corresponding to the two task types) per participant, whereas the other two rules are subsequent to aggregation over functional imaging runs and thus only vary in participant.

Neuroimaging processing steps can sometimes produce large numbers of output files (for example, Freesurfer’s recon-all command). Although listing each output path is recommended and is best practice, if the output files reside within a common parent directory and no other rules produce outputs within that directory, an alternative is to specify that directory as the rule’s output. When using this approach, the directory path is wrapped within the directory Snakemake helper function.

A processing step can also produce intermediate or extraneous files that are unnecessary for the workflow and do not need to be saved in the filesystem after the job has completed. Such files can be manually removed as part of the rule’s processing. However, a simpler approach is to use the shadow rule directive, which causes the rule to be executed in an isolated temporary location and only the listed output files copied to their destination after successful completion of the rule. The shadow directive is used in the temporal averaging and coregistration rules in the example workflow (Fig. 4) to discard ephemeral intermediate files. Using shadow is also useful for commands that create temporary files with fixed names during their processing, particularly when such files are created in the working directory; without shadow, parallel execution of rules can cause such temporary files to be read from and written to by multiple jobs—potentially with chaotic consequences.

### Inputs

Inputs are specified within the input directive and have similar characteristics to the outputs described above. A particularly useful approach to specifying inputs within a workflow is as a direct reference to the output of an upstream (parent) rule; indeed, it is a useful practice to aim to only ever specify the direct path for a given file at one location within the entire workflow. In our example workflow, the rules describe their inputs in terms of the output of a parent rule where possible.

Inputs can also be specified as Python functions that are evaluated when Snakemake constructs its representation of the workflow. Such input functions have a wildcards parameter, which has as its argument a Python object with wildcard variables as attributes. The function is required to return a single string, a list of strings, or a dictionary (with identifiers as keys and paths as values). For simple functions, anonymous (lambda) functions are often appropriate (see the rule shown in Fig. 4A).

Input functions are particularly useful for rules that need to aggregate over runs or participants. For example, the temporal averaging step in our example workflow requires inputs to comprise both functional imaging runs for a given participant. We thus only have one output wildcard: the participant identifier (sub_num). We can use an input function to return a two-item list of strings that describes the required input paths, given a particular value of sub_num (Fig. 4A). The Snakemake helper function collect is useful for inserting such values into strings containing wildcards.

### Mechanism

A Snakemake rule can be considered as a contract that a certain set of output files will be produced from a certain set of input files. The *mechanism* of the rule describes how this computational process is implemented. If the process is simple, it can be described using a shell directive in which the value contains the command string to execute. For example, the rule for temporal averaging in our example workflow is executed via the sequential execution of two AFNI commands (Fig. 4A). When specifying the shell command, attributes of the rule such as input and output can be referenced within the command to be replaced with the relevant value by Snakemake upon execution.

For any process that is more complicated or difficult to express in a shell command, it is preferable to delegate the description of the mechanism to an external script. Such scripts are typically saved within the scripts workflow sub-directory and can be written in programming languages such as Python, R, Julia, or Rust—or even Jupyter notebooks. The script is specified using a script directive in which the value is a path to the script file, relative to the location of the rule definition. During execution, the script has access to a variable that exposes the information contained within the calling rule. Although sufficiently simple that the shell directive would have been appropriate, we illustrate the use of the script directive in our example workflow in the motion correction step (Fig. 3).

The commands that are executed as part of a rule can often produce textual output that is useful for debugging and quality control. Snakemake provides the log directive to allow a path to be specified for capturing such output streams. This path can be used as the target for redirecting standard output and/or standard error either in the shell directive, as in the temporal averaging and coregistration rules, or within the processing script, as in the motion correction rule.

It is also common to require the use of external scripts for languages and applications that are not directly supported by Snakemake (e.g., a Matlab script). Here, a useful approach is to save the script inside the resources workflow sub-directory and register this path as an input item for the rule. Then, the contents of the script can be passed to the Matlab executable’s standard input within a shell command or an external script in a language like Python. By using environment variables, rule parameters can be interpreted from within the Matlab script (using Matlab’s getenv function).

An optional, but highly recommended, Snakemake capability is to execute jobs in containers. A container combines software and an operating system into a single unit (called an image) that can be executed by a container platform, with Docker being perhaps the most well-known (see Wiebels and Moreau, 2021, for a tutorial on containers in a research context). Snakemake uses Apptainer as its container platform, which is particularly suited to academic and high-performance computing. The advantage of using containers is the increase in reproducibility that is provided by the lack of a need to install the required software (often with a specific version requirement) and the ability to run the software in an isolated and ephemeral environment. Containers are specified using the container directive in the rule, with the value being any URL that is supported by Apptainer as the source of a container image.

Container images for common neuroimaging software can be obtained from the Neurodesk project (Renton et al. 2024). The available software can be viewed on the Neurodesk Github repository,^7^ which then links to images stored in the Docker package registry on Github. Such URLs can then be provided as the value for the container directive; for example, each rule in our example workflow specifies a URL from the Neurodesk project for AFNI. For software that is not explicitly packaged in a container by Neurodesk, custom containers can be created by using the visual interface in the Neurodesk interactive container builder^8^ or by creating Apptainer definitions (see Nüst et al., 2020) and building a container manually.^9^ Our accompanying website gives further details on the use of containers and provides an example of custom container creation.^10^

### Parameters

The commands that are executed within rules often have additional parameters that modify the operation of the command. Such parameters can sometimes be hard-coded within the rule’s mechanism (via the shell or script directive described above); however, to provide additional visibility and flexibility of such parameters, they can also be specifically named in the rule by using the params directive. For example, the motion correction step in the example workflow requires the ‘base’ image (the motion correction target) to be specified. By including the base item within the params directive, the rule’s mechanism then has access to params.base when building the command (Fig. 3B).

Parameters that apply across multiple rules or where it is desirable to be easily user-modified can also be specified within a global configuration. This configuration can be read from a file (in YAML or JSON format) at a location specified by the configfile directive. Alternatively, configuration settings can be provided at runtime using the –config parameter in the Snakemake launcher. Rules can then access the values of configured keys using standard Python dictionary notation on a variable named config.

### Resources

Specifying the computational requirements within a rule can assist with appropriate job scheduling and runtime behaviour. Perhaps the most useful such requirement is for RAM usage, which is specified by the mem key within an item in the resources directive of the rule. For example, specifying that executing a job from the rule is likely to use 5 GB of RAM allows Snakemake to schedule parallel jobs such that the RAM budget of the computational infrastructure is not exceeded. Similarly, the runtime key can be used to indicate the maximum time that a job would take to complete; this is particularly useful for cluster-based execution where such information typically needs to be provided to the cluster scheduler. Other standard resources that can be provided are disk (the required amount of disk space), gpu (the number of GPUs required), and tmpdir (the location for temporary storage).

Workflows are typically run on computing platforms with multiple ‘cores’, which provides the capacity for parallel processing both within and between jobs. The threads directive in a rule sets the number of cores that are allocated to a job that is executed from the rule; the default is one thread. Increasing the thread count can be useful to speed up the execution of programs that utilise multi-threading, such as some of the AFNI programs^11^; for example, we set the thread count in the coregistration rule to two (Fig. 4B) to allow programs called by align_epi_anat.py to use two cores. In addition to such within-job parallelisation, Snakemake also provides between-job parallelisation by executing multiple jobs simultaneously when the total number of threads across running jobs does not exceed the number of available cores; for example, a four-core computing environment would allow Snakemake to execute two coregistration jobs concurrently.

## Helper functionality

**Fig. 5:**
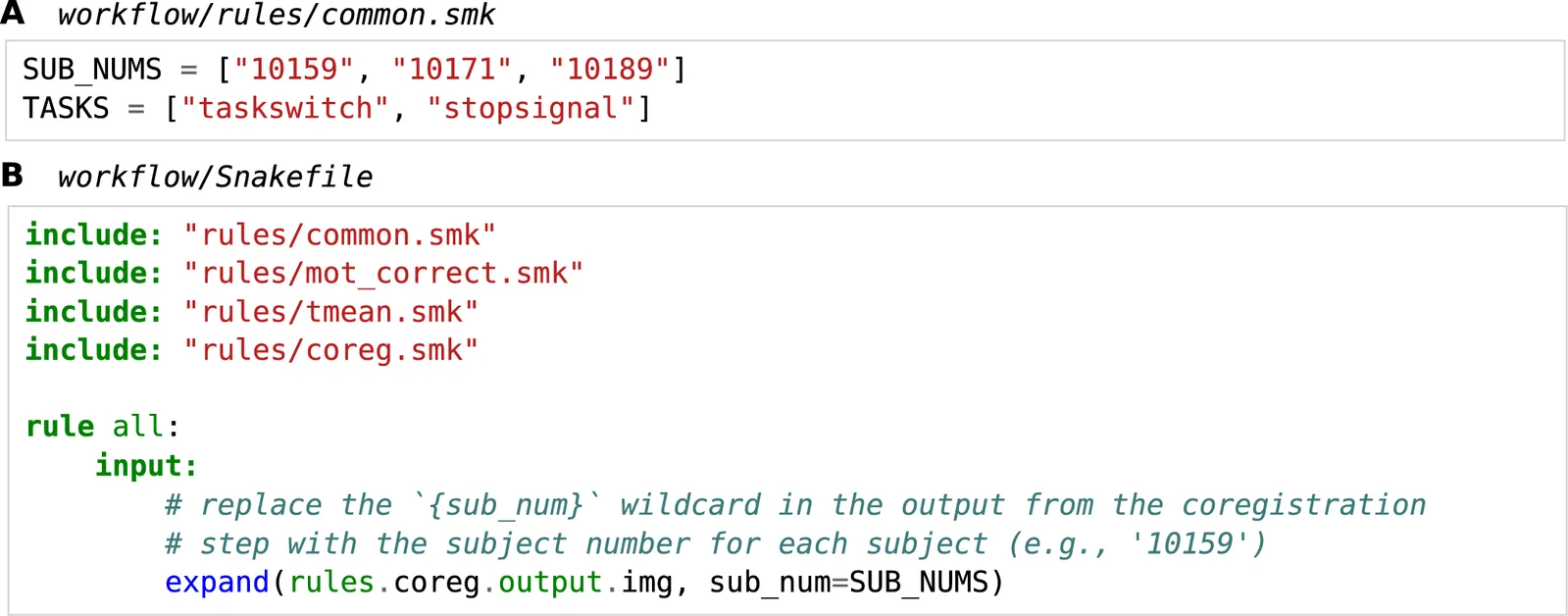
The rule containing helper functionality (**A**) and the Snakefile (**B**) for the example workflow

Neuroimaging workflows can require additional computational logic to be able to fully specify the details of the workflow. By convention, such logic is expressed in Python within a common.smk file in the rules directory. Definitions within this file are contained within a global namespace that is visible to the other rule files. In our example workflow, the common.smk file contains two constants that define the participant identifiers and the task names; this is shown in Fig. 5A, and a reference to the task names variable can be found in Fig. 4A.

For workflows where the raw data is in the Brain Imaging Data Structure (BIDS) format (Gorgolewski et al. 2016), the Python package pybids (Yarkoni et al. 2019) is very useful for parsing and accessing the metadata of the project input within the common.smk file. The snakebids package^12^ can also be used to simplify the process of specifying paths in BIDS format (while also providing additional functionality, such as the creation of BIDS applications).

Snakemake also contains a number of useful built-in helper functions, and their use is preferable, where possible, to specifying custom functions in common.smk or creating complex anonymous functions within rules. For example, it is useful for the coregistration rule to have a representation of the stem of the output filename (i.e., the path without its directory or suffix). This functionality is provided within the subpath helper function from Snakemake, as shown in Fig. 4B.

## Snakefile

The entry point for the Snakemake execution is a file named Snakefile (no extension). Within this file, the include directive is used to import each of the rule definition files. This importing operates sequentially, and the rule definition files are thus imported in an order that ensures that any cross-references between rules are defined at the time of importing.

The first rule (and often the only rule) in the Snakefile is, by default, the ‘target’ of Snakemake execution. By convention, this rule is named all. It is unusual in two main respects. First, it typically only contains an input directive. Second, each path within the input directive must not contain any wildcards.

Conceptually, the target rule can be thought of as specifying all the files that are going to be produced by the workflow but are not required by any other rules within the workflow. Because the rule requires each file to exist for it to operate as input, Snakemake then calculates which rule can be executed (given wildcard values extracted from the input path) that will produce the required files. As the final stage of the workflow, the rule does not need to produce any output.

For our example workflow, the final output is the anatomical image that is saved from the attempt to coregister the anatomical image with the functional images (we say ‘attempt’ to emphasise that such coregistration would always require verification in a complete workflow), for each participant. Hence, our all rule has as its input a reference to the output of the coregistration rule. However, we need to replace the wildcard placeholders in the coregistration rule with concrete values derived from our participant identifiers; we do this using the expand Snakemake helper function. For example, the first participant identifier is "10159" and so the first input path in the all rule will contain this value in its path. Upon execution, Snakemake will calculate that this input file can be produced if it runs the coregistration rule with "10159" as the value for the sub_num wildcard. It then calculates that the input to the coregistration rule can be produced if it runs the temporal averaging rule with "10159" as the value for the sub_num wildcard, and so on until it reaches an input file that exists outside of the workflow (the raw data). The Snakefile for the example workflow is shown in Fig. 5B.

## Workflow execution

A Snakemake workflow is executed using the snakemake command-line interface, typically from within the workflow’s root directory (the parent of the workflow directory). We recommend the liberal use of the –dry-run argument when interacting with the workflow; this prints the status of the workflow and the details of each of the jobs that Snakemake would execute, without actually running the workflow. For our example workflow, in which we specify containers for the execution of each rule, we need to activate the use of containers via the –use-apptainer argument. If we are running the workflow on a local machine, we also need to specify the number of cores that Snakemake is allowed to use (e.g., –cores all). We may also want to set the available memory that Snakemake is allowed to use; for example, we could allow 8 GB of RAM by the argument –resources mem_mb=8000.

As evident above, running Snakemake in a given computational environment can involve specifying a number of command-line arguments. This can be simplified by the use of ‘profiles’; a profile consists of a YAML file that expresses the arguments that would otherwise be supplied via the command-line. Such profiles are typically tied to the computational environment rather than to the workflow, and are hence stored outside of the workflow project in a system configuration location. However, profiles can also exist within a workflow for any settings that are critical to the operation of the workflow across computational environments. See our accompanying website^13^ for an example of the use of profiles.

Profiles are particularly useful when requesting that Snakemake execute jobs on a remote server. This is achieved via the use of ‘executor plugins’, which are specific to the remote computational environment (e.g., SLURM; Jette and Wickberg, 2023) and are installed separately from Snakemake.^14^ Such plugins can also be used to recruit different storage providers for the files (inputs and outputs). These ‘storage plugins’ are also installed separately to Snakemake and include options like S3 and sFTP. A particularly appealing aspect of this plugin architecture is that no changes to the rules are generally required; different computational platforms or storage providers can be applied by launching Snakemake with different arguments.

If a Snakemake workflow is successfully executed and the same Snakemake command is immediately re-run, there will typically be no need for any jobs to be launched. This is because Snakemake tracks the existence and provenance of rule components and only executes jobs that activate one or more ‘triggers’ that indicate that the workflow output is out of date. These triggers include file-related properties such as whether the modification time for a rule’s input file is later than the modification time for the rule’s output file and rule-related properties such as a change to the input, params, container, and shell directives and changes to the contents of the file referred to in the script directive. Because only those rules that require re-execution are actually executed, this Snakemake capability makes the development and revision of workflows to be computationally efficient and increases the validity of the outputs (e.g., by not forgetting to re-run upstream steps that are affected by a change).

**Fig. 6:**
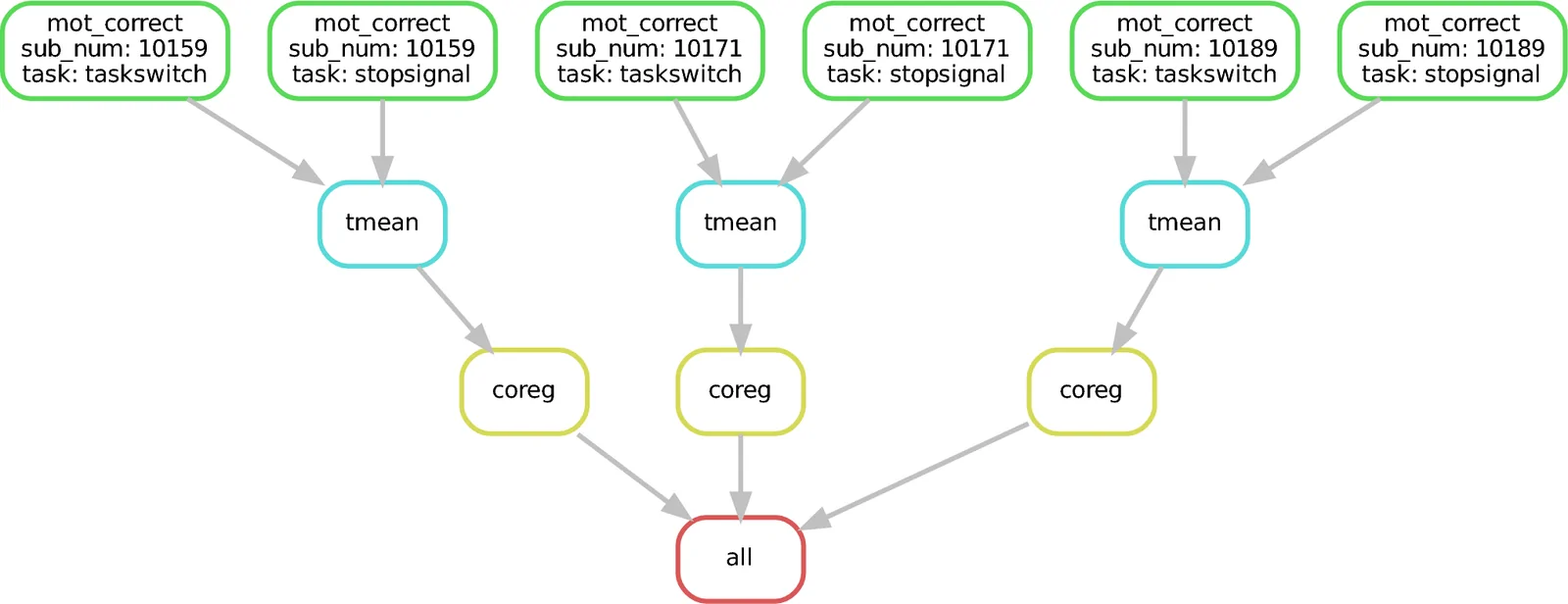
Visualisation of the example workflow as a directed acyclic graph (DAG), as produced by snakemake –dag

The snakemake command can also be used to generate a visualisation of the workflow. By providing either –dag, –filegraph, or –rulegraph arguments, Snakemake forms a graph of the dependency relationships between the rules in the workflow. The default is for the output to be produced in Graphviz^15^ format (in the plain-text DOT language), which can then be passed as input to the dot command (assuming Graphviz is installed on the system) to be rendered into an image; see Fig. 6 for the visualisation produced using the –dag option on the example workflow. This is particularly useful as a debugging and quality-control strategy, to ensure that the flow of data between processing steps is as anticipated.

## Example workflow summary

Through the above process, we have specified a workflow in the Snakemake framework and have seen how the workflow can be executed. The workflow specification has required some effort; we have introduced a new layer of abstraction (Snakemake), with its added dependencies, domain-specific language, and unfamiliar behaviour. Indeed, viewing the relative complexity of the contents of the files shown in Figs. 3–5 versus the small set of commands that are executed may make the overhead of this new layer not seem worthwhile. However, this perspective does not take into account the gains in interpretability, efficiency, and reproducibility that are obtained through the Snakemake framework—gains that become more salient as the workflow grows towards the complexity of realistic neuroimaging processing workflows. For example, any user with access to the workflow and its relevant raw data can reproduce its outputs by running a single command, with tolerance to their specific computational environment. The workflow can also form the basis of future workflows, with many rules and scripts likely to require only minimal editing.

## Additional recommendations for Snakemake usage

The process of implementing a workflow can be daunting due to the large number of rules, scripts, and dependencies that are required for the complexity of a complete neuroimaging processing workflow. We recommend adopting an iterative and modular approach, which conforms well to both the structure of typical neuroimaging processing workflows and to the approach of Snakemake. We suggest beginning with the first processing operation that depends on the raw data, and undertaking the following steps: 1) write the rule and any associated script (it is also often useful to use the benchmark directive in the rule to save a summary of the rule’s resource usage); 2) use the include directive to add the rule to the Snakefile, and set the input of the all rule to be the output of this rule with wildcards resolved to only a subset of their possible values (e.g., a single participant, possibly also a single run); 3) run Snakemake to execute the rule, monitoring for any error messages in the Snakemake output or the log files, and then evaluate the output files for conformance to the desired processing operation; 4) adjust the rule as necessary to implement the desired processing logic (and, potentially, to add or update resource requirements), and repeat the Snakemake execution and output evaluation. Once this rule is complete, the implementation can then move onto the next processing operation and the above steps are repeated—with the output of the existing rule forming the input for the new rule and the output of the new rule forming the input for the all rule. This iterative approach is particularly efficient in Snakemake, given that it will not require the re-execution of previously-executed (unchanged) rules. We provide a step-by-step demonstration of this incremental workflow creation approach on our accompanying website.^16^

For a researcher with an established set of processing operations, the conversion of such operations into the rules of a Snakemake workflow typically aligns with the intuitive separation of processing ‘steps’. However, there is some flexibility in the granularity of decomposition. For example, motion correction in fMRI is often performed in two sequential stages: within-run and then between-run. These stages could be implemented within a single rule (implementing both stages) or across two rules (one for within-run and one for between-run); one rule is perhaps simpler and reduces the overall number of distinct jobs to be run, but two rules facilitates parallel execution across runs. A further consideration for decomposition into rules is the software requirements. For example, a rule that uses AFNI for a subset of its functionality and SPM for another subset of its functionality would typically benefit from separation into two rules that can then use AFNI-specific and SPM-specific containers—rather than needing to find or create a container that has both AFNI and SPM.

Using software with proprietary foundations, such as those based on Matlab, can require additional considerations. Often the licensing of such software is communicated using environment variables. The Snakemake directive envvars is useful for signalling that the execution of a workflow requires a particular environment variable to be set; for example, MLM_LICENSE_FILE for Matlab. Regarding Matlab, we also note that, in our experience, it tends to not obey the threads directive in Snakemake rules; we have found running the Matlab function maxNumCompThreads to be required to avoid having Matlab use all cores.

The description of workflows thus far has assumed that the entire workflow can be executed autonomously. However, there can be occasions in neuroimaging workflows in which manual intervention is required. For example, some workflows may have steps that involve the use of a point-and-click interface, such as studies investigating visual cortex in which the early visual areas are manually defined from retinotopic mapping acquisitions. This requires some steps of the workflow to be executed (all of those upon which the manual step is dependent upon), but some steps cannot be completed until after the manual intervention (all of those that depend upon the manual step). A potential approach in this circumstance is to define an additional rule in the Snakefile, below the all rule, that has as its input the full paths (without wildcards) that are required for the manual step. Snakemake can then be executed with this rule’s name as the target (rather than all); this will execute all the processing that is required for the manual step to be performed, while not executing the processing that depends on the output of the manual step being available. Once the manual step is completed, Snakemake can be executed as normal to complete the workflow. Alternatively, a conventional autonomous workflow can be preserved, with a rule for the manual step in which the script simply waits for the set of output files to appear in the filesystem (from the manual intervention) and then completes.

## Conclusion

The inherently complicated nature of neuroimaging processing workflows can be contained and managed by using the Snakemake workflow software. By describing the details of the workflow operations in Snakemake’s domain-specific language, the challenges of workflow interpretability, execution efficiency, and reproducibility can be delegated to Snakemake and thus abstracted from the neuroimaging researcher. For those interested in developing their own Snakemake workflows, we highly recommend consulting Snakemake’s comprehensive documentation^17^; Snakemake is sophisticated software with much functionality that we have not covered here. We also recommend the “Introduction to Containers and Snakemake for Reproducible Research”^18^ for a general practical tutorial on Snakemake and working through the detailed workthrough that is contained in our accompanying website.^19^ We believe that the adoption of such practices would be beneficial for the neuroimaging researcher and the broader scientific community.

## Data Availability

The example workflow is available at https://github.com/unimelbmdap/snakemake_paper_demo_workflow. The raw data that is used in the workflow can be obtained via the workflow available at https://github.com/unimelbmdap/snakemake_paper_demo_workflow_data.

## Footnotes

1 https://unimelbmdap.github.io/neuroimaging_snakemake_tutorial/figure.html.

2 We note that we have not played any role in the genesis or ongoing development of Snakemake; we are neuroimaging practitioners who have found Snakemake useful in our research.

3 https://github.com/unimelbmdap/snakemake_paper_demo_workflow.

4 https://unimelbmdap.github.io/neuroimaging_snakemake_tutorial.

5 https://neurodesk.org/.

6 Here, we assume the raw data is already present in this directory; see the accompanying website (https://unimelbmdap.github.io/neuroimaging_snakemake_tutorial/acquire.html) for a demonstration of how the raw data can be downloaded as part of the workflow.

7 https://github.com/orgs/neurodesk/packages.

8 https://neurodesk.org/neurocontainers-ui.

9 See our repository at https://github.com/unimelbmdap/neuroimaging_containers for a set of such definitions.

10 https://unimelbmdap.github.io/neuroimaging_snakemake_tutorial/figure.html#container.

11 See https://afni.nimh.nih.gov/pub/dist/doc/misc/OpenMP.html.

12 https://github.com/khanlab/snakebids.

13 https://unimelbmdap.github.io/neuroimaging_snakemake_tutorial/setup.html#set-default-snakemake-command-line-arguments.

14 See the Snakemake plugin catalog at https://snakemake.github.io/snakemake-plugin-catalog.

15 https://www.graphviz.org/.

16 https://unimelbmdap.github.io/neuroimaging_snakemake_tutorial.

17 https://snakemake.readthedocs.io.

18 https://sib-swiss.github.io/containers-snakemake-training/.

19 https://unimelbmdap.github.io/neuroimaging_snakemake_tutorial.
